# DNA Damage Response Alterations Stratify Response to ICI-Based Therapy in Advanced NSCLC with High PD-L1 Expression

**DOI:** 10.3390/curroncol33090508

**Published:** 2026-08-26

**Authors:** Fang Hao, Linlin Zhang, Diansheng Zhong

**Affiliations:** Department of Oncology, Tianjin Medical University General Hospital, Tianjin 300052, China

**Keywords:** non-small cell lung cancer, high PD-L1 expression, DNA damage response, clinical benefit

## Abstract

**Background**: Although high PD-L1 expression correlates with improved outcomes to immune checkpoint inhibitor (ICI), it remains an imperfect predictive biomarker. DNA damage response (DDR) pathway alterations are closely associated with antitumor immunity, shaping tumor immunogenicity and the immune microenvironment. This study evaluates the distribution and clinical impact of DDR alterations in advanced Non-small cell lung cancer (NSCLC) with Programmed death-ligand 1 (PD-L1) ≥ 50%, providing insights for personalized treatment selection and response prediction. **Experimental Design**: Patients with advanced NSCLC and PD-L1 expression ≥ 50% who received first-line ICI-based therapy were retrospectively enrolled. Tumor tissue samples underwent targeted next-generation sequencing using a comprehensive cancer panel, with a predefined 35-gene DDR panel used to identify and classify pathogenic or likely pathogenic DDR alterations. The associations between genomic DDR alteration status, clinicopathologic characteristics, cytokine profiles, and immunotherapy outcomes were evaluated. **Results**: Among 121 patients, 72 (59.5%) were classified as DDR-positive and 49 (40.5%) as DDR-negative based on genomic DDR alterations. DDR-positive patients exhibited a higher burden of DDR alterations and TMB, and DDR-positive status remained independently associated with improved outcomes after adjustment for clinical factors and TMB. DDR-positive patients had higher DCB rates and IL-2 levels, whereas TNF-α and IL-6 levels were elevated in DDR-negative patients. DDR-positive status was associated with longer PFS than DDR-negative status (median, 12.7 vs. 8.9 months), with the benefit of chemo-immunotherapy mainly observed in DDR-positive patients. **Conclusions**: Genomic DDR alterations are associated with distinct immune profiles and may represent a potential biomarker for immunotherapy stratification. DDR-positive patients may derive greater benefit from chemo-immunotherapy, supporting further prospective investigation.

## Simple Summary

Immune checkpoint inhibitors have improved outcomes for patients with advanced non-small cell lung cancer, particularly those with high programmed death-ligand 1 expression. However, a proportion of patients derive limited benefit from these treatments, highlighting the need for additional biomarkers to guide therapeutic decisions. In this study, we investigated genomic alterations in the DNA damage response pathway and their association with immunotherapy outcomes in advanced non-small cell lung cancer. Patients with multiple DNA damage response gene alterations showed distinct immune profiles, higher rates of durable clinical benefit, and longer progression-free survival. They also appeared to derive greater benefit from combined chemotherapy and immunotherapy. These findings suggest that genomic DNA damage response alterations may help identify patients who are more likely to benefit from personalized immunotherapy strategies.

## 1. Introduction

Non-small cell lung cancer (NSCLC), accounting for approximately 85% of all lung cancer cases, remains the leading cause of cancer-related deaths both in China and worldwide [1,2]. At the population level, advances in the identification of key genomic alterations, together with the advent of immune checkpoint inhibitors (ICIs), have significantly improved 3-year relative survival and reshaped treatment paradigms [3]. Programmed death-ligand 1 (PD-L1), an immune checkpoint ligand expressed on tumor cells and multiple components of the tumor microenvironment, interacts with its receptor programmed death-1 (PD-1), which is primarily expressed on activated T cells. This PD-1/PD-L1 axis represents a fundamental mechanism of immune suppression by attenuating T-cell activation and enabling tumor immune escape [4]. PD-L1-targeted and PD-1-targeted monoclonal antibodies, including atezolizumab, durvalumab, nivolumab, and pembrolizumab, have demonstrated remarkable clinical efficacy across diverse malignancies and have become a cornerstone of modern cancer immunotherapy [5]. High PD-L1 expression, defined as a tumor proportion score (TPS) ≥ 50%, represents an established biomarker associated with increased clinical benefit from ICIs. In the phase III KEYNOTE-024 trial, first-line pembrolizumab improved clinical outcomes compared with platinum-based chemotherapy in patients with advanced NSCLC and TPS ≥ 50% [6]. Similar survival benefits were reported in subsequent phase III trials, including KEYNOTE-042 and IMpower110 [7,8]. However, PD-L1 expression alone does not adequately reflect the heterogeneity of immune responses to ICIs. Additional biomarkers, such as tumor mutational burden (TMB), microsatellite instability (MSI), and immune-related gene expression signatures, may provide complementary information for patient selection.

DNA damage response (DDR) pathway alterations have been increasingly linked to sensitivity to immunotherapy, with potential implications for tumor–immune interactions and patient selection [9]. In lung cancer, DDR alterations are relatively common and have been associated with tobacco-related mutagenesis and higher tumor mutational burden (TMB) [10,11]. Genomic instability resulting from defective DNA repair can contribute to tumor development while also creating potential therapeutic vulnerabilities [12,13,14]. DDR activity can influence antitumor immunity through effects on tumor immunogenicity, the tumor immune microenvironment, and immune checkpoint signaling [15]. Loss of DDR function may increase tumor vulnerability to synthetic lethal approaches and enhance immune recognition in some settings [12]. These interactions provide a rationale for investigating DDR alterations as potential biomarkers and for exploring DDR-directed combination strategies with immunotherapy.

Given the persistent heterogeneity of therapeutic response among patients with PD-L1-high NSCLC and the potential impact of DDR alterations on treatment sensitivity, we performed a single-center retrospective analysis of genomic mutation data to characterize the clinical and molecular features of DDR gene alterations in this population. Our aim was to define the DDR alteration landscape and evaluate its clinical relevance, thereby providing insights into treatment response heterogeneity and the development of personalized therapeutic strategies.

## 2. Materials and Methods

### 2.1. Patient Recruitment

**Figure 1:**
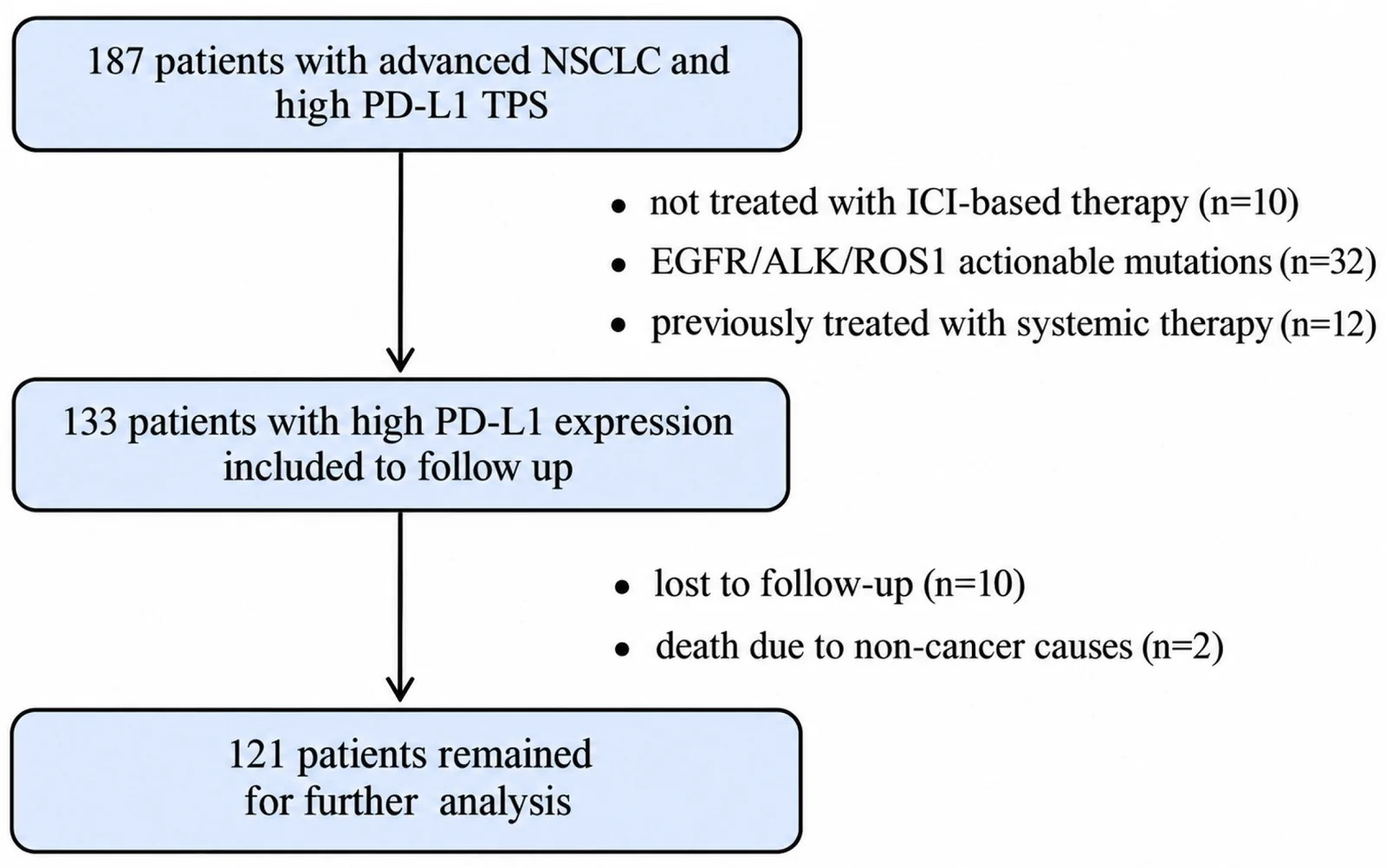
Overview of patient selection. A total of 187 patients with NSCLC were initially screened, of whom 133 patients with high PD-L1 expression were subsequently identified and enrolled. Among these, 2 patients died from non-cancer-related causes and 10 were lost to follow-up. Ultimately, 121 patients were included in the final analysis cohort. Non-small cell lung cancer: NSCLC; programmed death-ligand 1: PD-L1; tumor proportion score: TPS; immune checkpoint inhibitor: ICI); epidermal growth factor receptor: EGFR; anaplastic lymphoma kinase: ALK; and ROS proto-oncogene 1, receptor tyrosine kinase: ROS1.

This exploratory, single-center study retrospectively reviewed 187 patients with a PD-L1 TPS ≥50% who were treated at our Department of Oncology between December 2022 and April 2025 (Figure 1 illustrates the cohort selection process). The key inclusion criteria were as follows: (i) histologically confirmed advanced NSCLC with available PD-L1 testing results; (ii) receipt of first-line ICI-based therapy, administered either as monotherapy or in combination with platinum-based chemotherapy; and (iii) absence of sensitizing driver alterations, including epidermal growth factor receptor (*EGFR*) mutations, anaplastic lymphoma kinase (*ALK*) rearrangements, and ROS proto-oncogene 1, receptor tyrosine kinase (*ROS1*) fusions. After applying the predefined eligibility criteria, 121 patients were ultimately included in the final analysis.

### 2.2. Patient Variables Collection

The collected data included age, sex, smoking status, histologic subtype, PD-L1 expression, TMB, genomic alterations, and baseline cytokine levels. Histologic subtypes included sarcomatoid carcinoma, adenocarcinoma and squamous-cell carcinoma. This retrospective study was approved by the Ethics Committee of Tianjin Medical University General Hospital. The requirement for informed consent was waived due to the retrospective nature of the study and the use of de-identified patient data. The study was conducted in accordance with the Declaration of Helsinki.

### 2.3. Tissue-Based Next-Generation Sequencing and Genomic Data Analysis

Genomic DNA extracted from formalin-fixed, paraffin-embedded tumor samples was subjected to comprehensive genomic profiling using a targeted sequencing panel covering DDR-related genes. The average sequencing depth exceeded 250×, with more than 100× coverage achieved across over 99% of exons. Genomic alterations, including deletions, truncations, and loss of function events such as nonsense mutations, homozygous loss, frameshift mutations, and intragenic rearrangements, were identified using tissue based next generation sequencing. All candidate variants were subsequently subjected to manual review using the Integrative Genomics Viewer.

TMB was calculated based on the entire coding region covered by the comprehensive genomic profiling assay and was defined as the number of nonsynonymous somatic mutations per megabase (Muts/Mb). Synonymous variants, non-coding variants, germline variants, and variants with insufficient sequencing support were excluded from TMB calculation. PD L1 expression was evaluated by immunohistochemistry using the PD L1 IHC 22C3 pharmDx assay from Dako (Glostrup, Denmark) and was quantified as the TPS according to the manufacturer’s instructions.

$$TPS\left(\%\right)=\frac{No.PD-L1-stained\;tumor\;cells}{Total\;No.\;of\;viable\;tumor\;cells}\;\times \;100$$

TMB was calculated using the entire coding region covered by the 825-gene panel, whereas DDR alterations were assessed using a predefined 35-gene panel covering eight major DNA repair pathways, including base excision repair (BER) (*MUTYH*, *PARP1*), damage sensor (DS) (*ATM*, *ATR*, *CHEK1*, *CHEK2*), Fanconi anemia (FA) signaling pathway (*FANCA*, *FANCC*, *FANCG*, *FANCL*, *XRCC*), homologous recombination (HR) (*BARD1*, *BRCA1*, *BRCA2*, *BRIP1*, *NBN*, *PALB2*, *RAD51*, *RAD51B*, *RAD51C*, *RAD51D*, *RAD52*, *RAD54L*), mismatch repair (MMR) (*MLH1*, *MSH2*, *MSH3*, *MSH6*, *PMS2*), nucleotide excision repair (NER) (*ERCC4*, *POLE*), SWI/SNF chromatin remodelling (*ARID1A*, *SMARCA4*), and TP53 pathway (*MDM2*, *MDM4*, *TP53*).

### 2.4. Plasma Cytokine Profiling

Peripheral blood samples were collected at baseline before initiation of first-line ICI-based therapy. Plasma was separated by centrifugation and stored at −80 °C until analysis. Circulating interleukin-2 (IL-2), interleukin-6 (IL-6) and tumor necrosis factor-alpha (TNF-α) levels were quantified using a multiplex bead-based immunoassay (Luminex platform) according to the manufacturer’s protocol. All measurements were performed in duplicate, with concentrations calculated using recombinant cytokine standard curves and validated by internal quality controls.

### 2.5. Evaluated End Points

All patients who underwent at least one follow-up radiologic tumor assessment were included in the efficacy analysis. Tumor response was assessed by the treating physician according to Response Evaluation Criteria in Solid Tumors (RECIST) version 1.1. Durable clinical benefit (DCB) was defined as a progression-free survival (PFS) duration of ≥6 months from treatment initiation, whereas patients with disease progression or death within 6 months were classified as having no durable benefit (NDB). PFS was calculated from the initiation of treatment to the date of radiographic disease progression or death from any cause.

### 2.6. Statistical Analysis

Statistical analyses were conducted with GraphPad Prism (version 9.5.0) and R (version 4.4.1). Baseline characteristics were described using descriptive statistics. Continuous variables were reported as medians with ranges, and categorical variables were summarized as frequencies and percentages. Group comparisons were performed using the chi-square test or Fisher’s exact test, as appropriate. Survival distributions were estimated using the Kaplan–Meier method and compared with the log-rank test. Cox proportional hazards regression analyses were performed to estimate hazard ratios (HRs) and 95% confidence intervals (CIs). Multivariable Cox models were constructed based on clinically relevant variables, including DDR status, treatment regimen, age, ECOG performance status, smoking history, PD-L1 expression level, and TMB. All statistical tests were two-sided, with *p*-value < 0.05 considered statistically significant.

## 3. Results

### 3.1. Clinical Characteristics of the Patients

Given the complexity and functional heterogeneity of the DDR network, alterations in a single DDR-related gene may not necessarily indicate clinically meaningful disruption of DNA repair capacity. Therefore, in this exploratory analysis, DDR-positive status was defined as the presence of deleterious somatic alterations affecting two or more DDR-related genes. This classification was intended to represent a composite genomic phenotype of DDR pathway disruption rather than a direct measure of functional DDR deficiency. Deleterious alterations were defined as pathogenic or likely pathogenic variants, including protein-truncating alterations (nonsense mutations and frameshift insertions/deletions), canonical splice-site variants, and previously reported deleterious alterations. Variants of uncertain significance and variants without established functional relevance were excluded. Patients harboring alterations in fewer than two DDR-related genes were classified as DDR-negative.

The main clinicopathological characteristics stratified by DDR mutation status are summarized in Table 1. A total of 121 patients with high PD-L1 expression were included, of whom 72 (59.5%) were classified as DDR-positive and 49 (40.5%) as DDR-negative. In the DDR-positive group, the median age at diagnosis was 67 years (range, 44–85), and most patients were male (77.8%) with adenocarcinoma (59.7%), with a comparable distribution observed in the DDR-negative group. Similarly, no significant differences were observed between the two groups in smoking status, biopsy site, baseline metastatic burden, lymph node stage, or immunotherapy regimen. Among DDR-positive patients, 46 (63.9%) had a progression-free survival of at least 6 months and achieved DCB, representing a significantly higher rate than in the DDR-negative group (63.9% vs. 42.9%, *p* = 0.026), while NDB was more common in the DDR-negative group. In addition, DDR-positive patients were more likely to have elevated TMB levels (≥10 mutations/Mb) than DDR-negative patients (34.7% vs. 14.3%, *p* = 0.013), suggesting enhanced tumor immunogenicity associated with DDR alterations.

### 3.2. DDR Gene Alteration Profile in PD-L1-High NSCLC

Table 2 summarizes the distribution of DDR gene alterations across functional repair pathways and their association with clinical benefit. DDR alterations were observed across multiple repair pathways, including base excision repair, damage sensing, homologous recombination, mismatch repair, nucleotide excision repair, Fanconi anemia, chromatin remodeling, and the TP53 pathway. Notably, alterations in the TP53 pathway were the most frequent in both groups, with TP53 mutations detected in 63.9% of patients in the DCB group and 73.5% of patients in the NDB group, suggesting a high prevalence but limited discriminatory value for clinical benefit stratification.

Within the SWI/SNF chromatin remodeling pathway, ARID1A mutations were enriched in the DCB group (18.1% vs. 4.1%). Similarly, alterations in Fanconi anemia pathway genes, particularly *FANCA* (11.1% vs. 2.0%), were more frequently observed in patients achieving DCB. In contrast, mutations in damage sensor genes, such as ATR (11.1% vs. 22.4%) and *CHEK2* (1.4% vs. 6.1%), were more prevalent in the NDB group, indicating a possible link with inferior clinical outcomes. Alterations in homologous recombination repair genes, including *BRCA1*, *BRCA2*, and *PALB2*, were detected in both groups, with a modest enrichment of *PALB2* mutations in the DCB group (8.3% vs. 2.0%). Meanwhile, genes involved in mismatch repair and nucleotide excision repair pathways showed relatively low mutation frequencies, without a clear pattern of association with clinical benefit.

### 3.3. Patterns of DDR Gene Alterations and TP53 Mutations in DCB and NDB Groups

**Figure 2:**
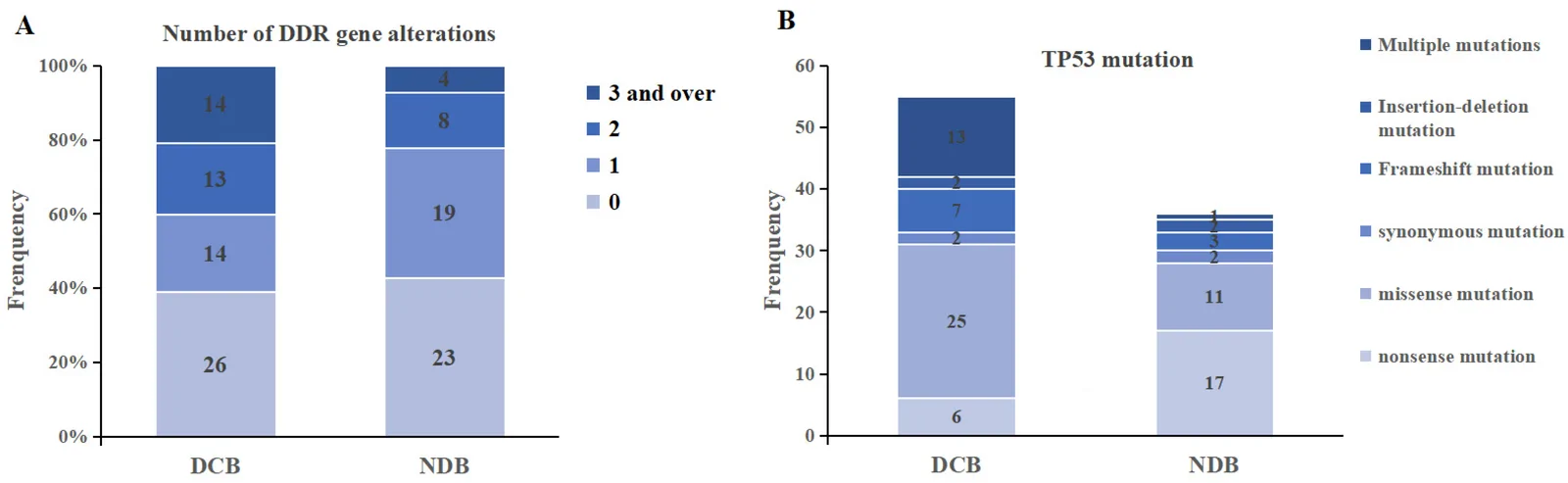
Distribution of non-*TP53* DDR alteration burden (**A**) and *TP53* mutation patterns (**B**) according to durable clinical benefit status. TP53: tumor protein p53; DCB: durable clinical benefit; NDB: no durable benefit; DDR: DNA damage response.

The distribution of non-*TP53* DDR gene alteration burden according to durable clinical benefit status is shown in Figure 2A. Patients with DCB tended to harbor a higher burden of non-*TP53* DDR alterations, with a greater proportion exhibiting ≥2 non-*TP53* DDR alterations compared with those in the NDB group. Conversely, single or no non-*TP53* DDR alterations were more frequently observed among patients without durable clinical benefit. Notably, this analysis reflects the distribution of non-*TP53* DDR alteration burden and should not be interpreted as being equivalent to the predefined DDR-positive/DDR-negative classification used in subsequent prognostic analyses.

*TP53* mutation analysis revealed that missense mutations were the predominant subtype in both the DCB and NDB groups, followed by nonsense mutations. Other mutation subtypes, including frameshift mutations, insertions/deletions (indels), synonymous mutations, and multiple *TP53* mutation events, were observed at low frequencies in both cohorts (Figure 2B). The overall distribution of *TP53* mutation subtypes did not differ significantly between the two groups, suggesting that *TP53* mutation patterns alone have limited discriminatory value for distinguishing patients with or without durable clinical benefit.

### 3.4. Association of DDR Status with Cytokine Profiles

**Figure 3:**
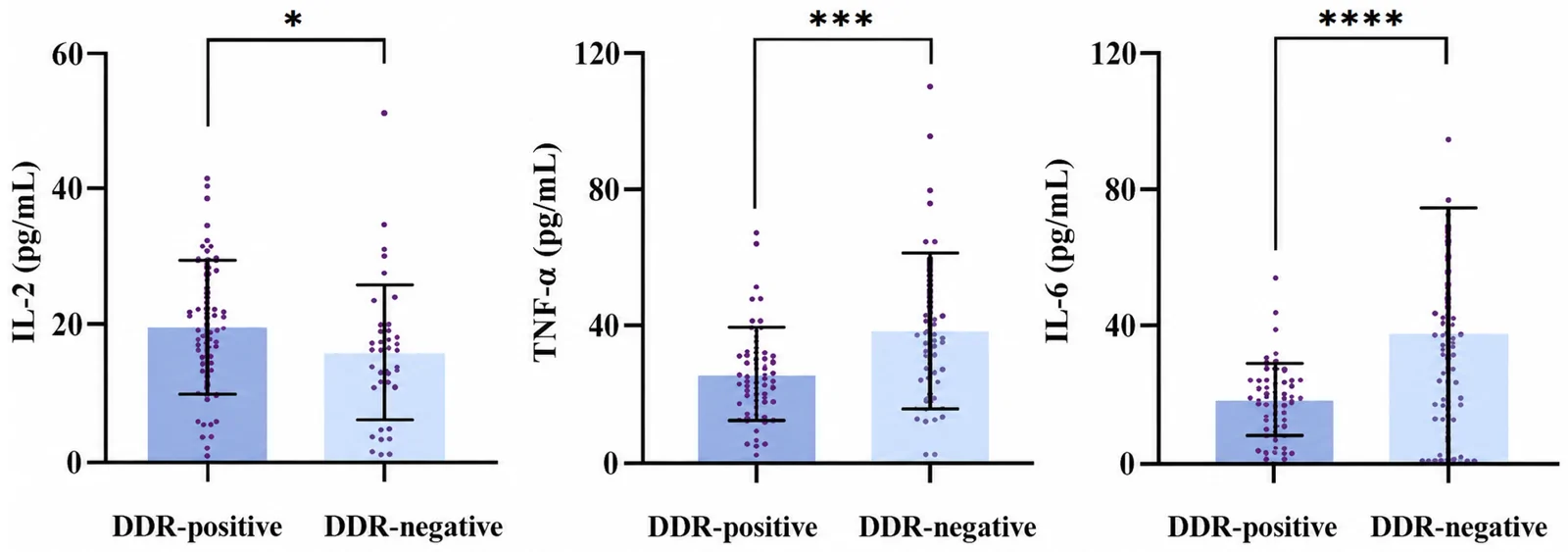
Comparison of baseline cytokine levels between DDR-positive and DDR-negative patients. Statistical significance is denoted as *p* < 0.05, * *p* < 0.05, *** *p* < 0.001, and **** *p* < 0.0001. IL-2: Interleukin-2; TNF-α: tumor necrosis factor-alpha; IL-6: Interleukin-6. DDR: DNA damage response.

Cytokine measurements were available for all 121 patients included in the cohort. Exploratory analysis of circulating cytokine profiles revealed differences according to DDR status (Figure 3). DDR-positive patients exhibited higher circulating IL-2 levels than DDR-negative patients (*p* < 0.05). Conversely, IL-6 and TNF-α concentrations were increased in the DDR-negative group, with more pronounced differences observed for IL-6 (*p* < 0.0001) and TNF-α (*p* < 0.001). These findings indicate an association between DDR status and systemic cytokine patterns; however, the biological implications of these peripheral immune alterations require further validation.

### 3.5. Treatment Efficacy Stratified by DDR Status and Treatment Regimens

**Figure 4:**
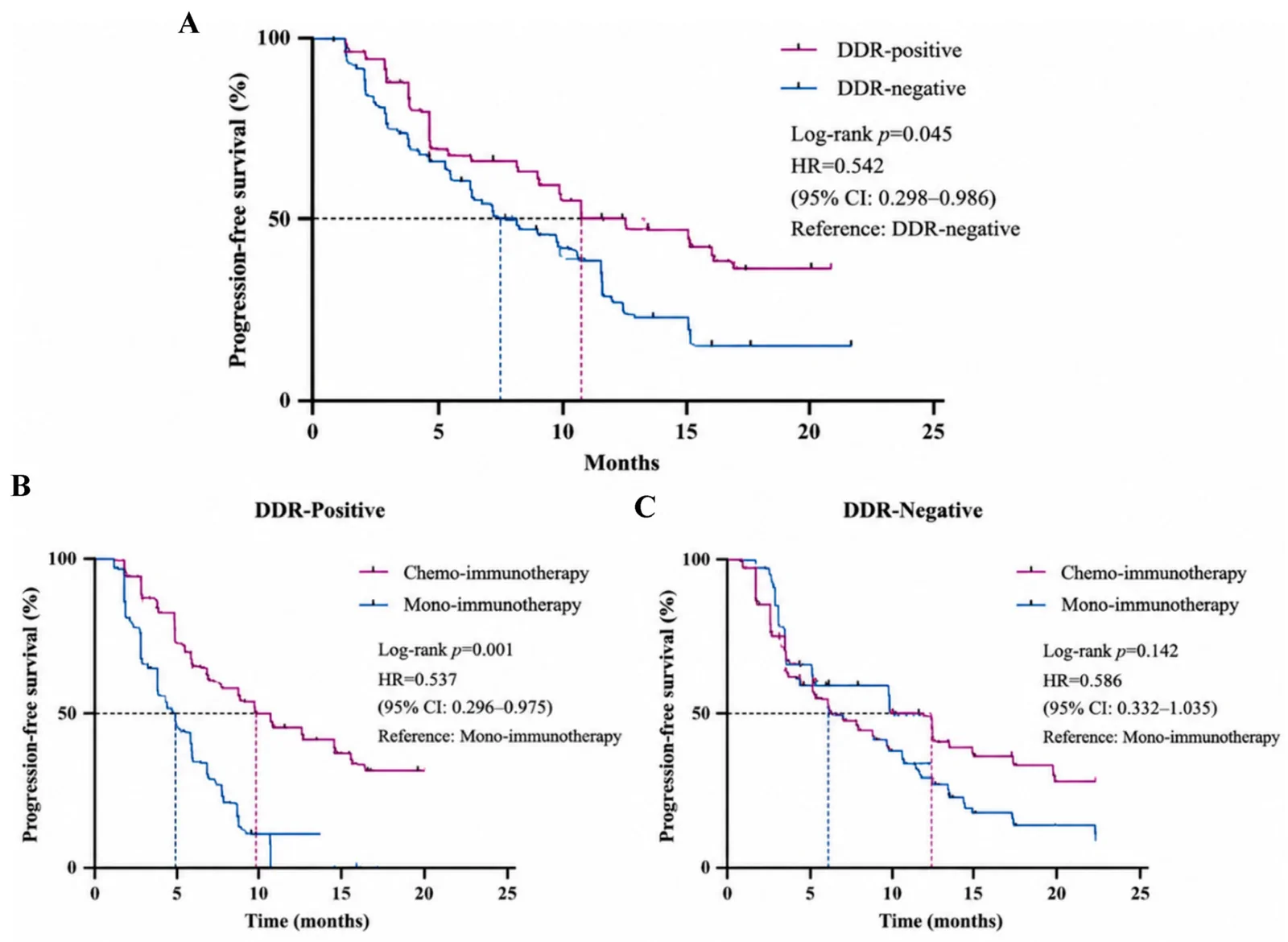
Association between DDR status, treatment strategy, and PFS. (**A**) DDR-positive patients showed longer PFS than DDR-negative patients (log-rank *p* = 0.045; HR = 0.542, 95% CI: 0.298–0.986; DDR-negative as the reference). (**B**) In the DDR-positive subgroup, chemo-immunotherapy was associated with improved PFS compared with mono-immunotherapy (log-rank *p* = 0.001; HR = 0.537, 95% CI: 0.296–0.975; mono-immunotherapy as the reference). (**C**) In the DDR-negative subgroup, chemo-immunotherapy showed a favorable trend in PFS compared with mono-immunotherapy, without statistical significance (log-rank *p* = 0.142; HR = 0.586, 95% CI: 0.332–1.035; mono-immunotherapy as the reference). HRs were estimated using Cox proportional hazards models with progression or death as the event. DDR: DNA damage response; PFS: progression-free survival; HR: hazard ratio.

Patients were stratified according to DDR status and treatment strategy (chemo-immunotherapy versus mono-immunotherapy) for subgroup analyses. In the overall cohort, DDR-positive patients demonstrated significantly longer PFS than DDR-negative patients (log-rank *p* = 0.045; HR = 0.542, 95% CI: 0.298–0.986), with median PFS of 12.7 and 8.9 months, respectively (Figure 4A). Among DDR-positive patients, chemo-immunotherapy was associated with improved PFS compared with mono-immunotherapy (log-rank *p* = 0.001; HR = 0.537, 95% CI: 0.296–0.975; Figure 4B). In the DDR-negative subgroup, chemo-immunotherapy also showed a numerically favorable association with PFS, although statistical significance was not reached (log-rank *p* = 0.142; HR = 0.586, 95% CI: 0.332–1.035; Figure 4C). The treatment-by-DDR interaction test was not statistically significant, indicating that the differential association between treatment strategy and PFS according to DDR status was not statistically confirmed.

To assess whether DDR status was independently associated with PFS beyond TMB, multivariable Cox regression analysis incorporating DDR status, TMB, treatment regimen, age, ECOG performance status, smoking history, and PD-L1 expression level was performed (Supplementary Figure S1). After adjustment for these covariates, DDR-positive status remained independently associated with prolonged PFS (HR = 0.54, 95% CI: 0.31–0.94, *p* = 0.020; DDR-negative as the reference group), whereas TMB was not significantly associated with PFS (HR = 0.75, 95% CI: 0.45–1.25, *p* = 0.262). These findings suggest that DDR alterations may provide complementary biological information beyond TMB and represent an independent biomarker associated with PFS in patients with PD-L1-high NSCLC.

## 4. Discussion

The relationship between DDR pathways and treatment outcomes in advanced NSCLC has been primarily explored in the context of platinum-based chemotherapy rather than immunotherapy. Existing evidence suggests that DDR proficiency may influence therapeutic response, as exemplified by the predictive value of *ERCC1* codon 118 polymorphism for platinum sensitivity [16]. In contrast, *XPD* polymorphisms have not demonstrated similar predictive utility, highlighting the functional heterogeneity within DDR components and indicating that not all DNA repair genes contribute equally to treatment response. Despite these insights, few studies have specifically evaluated whether DDR alterations influence immunotherapy outcomes in patients with PD-L1 expression ≥ 50%, representing an important knowledge gap at the intersection of genomic instability and immune-based treatment selection. Mechanistically, genomic alterations affecting DDR pathways have been proposed to contribute to a more immunogenic tumor phenotype through increased tumor mutational burden and neoantigen generation, which may subsequently influence immune recognition and sensitivity to immune checkpoint inhibitors [17]. However, this hypothesis remains insufficiently validated in clinically defined PD-L1-high populations. In the present study, we comprehensively evaluated the associations among clinical outcomes, cytokine profiles, and DDR alteration status in patients with NSCLC and high PD-L1 expression. A targeted DDR gene panel comprising 35 genes across eight major DNA repair pathways was employed to characterize DDR alteration patterns within this clinically defined population. By integrating molecular and clinical characteristics, our findings provide insights into the biological heterogeneity of PD-L1-high NSCLC and highlight the potential association between DDR alterations, immune-related features, and treatment response.

Single-agent pembrolizumab has become the preferred first-line therapy for patients with advanced NSCLC and PD-L1 expression ≥ 50%, marking a paradigm shift from the platinum-based chemotherapy doublets that historically constituted the standard of care [6,7]. This treatment recommendation applies uniformly across both non-squamous and squamous cell carcinoma histologies, suggesting that PD-L1 expression represents a more powerful predictor of immunotherapy benefit than traditional histological classification [18,19]. However, a substantial proportion of patients with PD-L1-high NSCLC do not derive durable clinical benefit, suggesting that PD-L1 alone is insufficient to capture the full complexity of tumor-immune interactions. Moreover, the inclusion of multiple alternative strategies in clinical guidelines, particularly combination chemoimmunotherapy regimens, further indicates that PD-L1 ≥ 50% status alone does not definitively determine optimal treatment selection [20]. The availability of pembrolizumab combined with carboplatin and pemetrexed, atezolizumab-based triplet or quadruplet regimens, and other combinations suggests ongoing uncertainty about whether all patients with high PD-L1 expression benefit equally from monotherapy versus combination approaches.

Several mechanistic hypotheses suggest DNA repair pathway status might influence immunotherapy response [21]. Alterations affecting DNA repair pathways may increase tumor mutational burden through impaired correction of replication errors and accumulation of unrepaired DNA damage [22]. In our cohort, DDR-positive patients were more likely to exhibit a high TMB (≥10 mutations/Mb) than DDR-negative patients, consistent with previous evidence linking alterations in DDR pathways to increased genomic instability and mutational burden. Higher TMB may contribute to increased neoantigen generation and a more immunogenic tumor phenotype [23], which could partially explain the enhanced sensitivity to immune checkpoint blockade observed in some patients. Importantly, multivariable Cox regression analysis demonstrated that DDR-positive status remained independently associated with prolonged PFS after adjustment for TMB and other clinical variables, suggesting that the predictive value of DDR alterations may extend beyond their association with TMB. These findings indicate that DDR alterations may represent an additional biomarker for immunotherapy stratification in PD-L1-high NSCLC, although the underlying biological mechanisms require further investigation.

Interestingly, DDR-positive patients had higher IL-2 levels, whereas IL-6 and TNF-α levels were higher in DDR-negative patients. These differences may indicate variation in systemic immune activity according to DDR status. However, circulating cytokine levels can reflect peripheral immune activity but may not fully capture the immune conditions within the tumor microenvironment. Higher IL-6 and TNF-α levels in DDR-negative patients may indicate a more pro-inflammatory state [24]. Recent studies have also linked inherited DDR variants to systemic immune characteristics, including peripheral blood mononuclear cell (PBMC) immune profiles, suggesting a possible role of host genetic background in tumor–immune interactions [25]. In our cohort, the differences in circulating cytokines between DDR groups may reflect differences in the type of immune response rather than simply its magnitude.

From a biological perspective, PD-L1 expression reflects an adaptive immune resistance phenotype; however, it does not fully capture upstream genomic instability or downstream immune effector functions [15,26]. Therefore, integrating DDR-related biomarkers may provide additional biological information for patient stratification. In our cohort, patients achieving durable clinical benefit exhibited a higher frequency of multiple DDR gene alterations (≥2), and DDR-positive status was associated with prolonged PFS compared with DDR-negative status, suggesting a potential association between DDR alterations and improved clinical outcomes. Subgroup analyses further indicated that DDR-positive patients receiving chemo-immunotherapy experienced longer PFS than those receiving mono-immunotherapy. However, given the retrospective design and non-randomized treatment allocation, these findings should be interpreted cautiously and do not establish DDR status as a validated predictive biomarker for treatment selection. Rather, DDR alterations may represent a genomic context associated with a potentially more immune-responsive phenotype and greater benefit from combination treatment strategies. Chemotherapy-induced remodeling of the tumor microenvironment, including increased antigen release and modulation of immunosuppressive components, may partially contribute to these observations. Prospective studies are warranted to further determine the clinical utility of DDR status in guiding immunotherapy-based treatment strategies.

Our study has several limitations. First, the relatively small sample size may limit the generalizability of our findings. Second, our analysis was based on peripheral cytokine levels, which may not fully reflect the tumor microenvironment. Tissue-based immune profiling, such as multiplex immunohistochemistry or single-cell RNA sequencing, could help characterize the tumor immune microenvironment more directly. In addition, treatment-related toxicity was not directly assessed in our study, and the relationship between cytokine profiles and treatment safety therefore remains unclear. Finally, prospective studies are needed to further evaluate the predictive value of DDR status and to determine whether DDR-guided treatment strategies can improve clinical outcomes.

In conclusion, our findings suggest that DDR alteration status is associated with distinct circulating cytokine profiles in patients with advanced NSCLC and high PD-L1 expression. Patients with DDR-positive tumors had higher circulating IL-2 levels, whereas those with DDR-negative tumors had higher IL-6 and TNF-α levels. DDR-positive status was also associated with longer PFS, while favorable outcomes were observed among patients receiving chemo-immunotherapy. These findings should be interpreted cautiously because of the retrospective design and non-randomized treatment allocation. Further prospective studies are needed to determine whether DDR status can be used to identify patients who are more likely to benefit from immunotherapy-based treatment.

## Figures and Tables

**Table 1:** Patient clinical characteristics.

Patient Characteristics	DDR-Positive (n = 72)	DDR-Negative (n = 49)	*p* Value
Median age, years (min–max)	67 (44–85)	66 (37–87)	0.332
Gender, n (%)			0.977
Male	56 (77.8%)	38 (77.6%)	
Female	16 (22.2%)	11 (22.4%)	
Smoking, n (%)			0.284
No	15 (20.8%)	15 (30.6%)	
past or current smoker	57 (79.2%)	34 (69.4%)	
Tumour biopsy site			0.841
Local	49 (68.1%)	35 (71.4%)	
Metastatic	23 (31.9%)	14 (28.6%)	
Histology, n (%)			0.522
sarcomatoid carcinoma	3 (4.2%)	3 (6.1%)	
adenocarcinoma	43 (59.7%)	26 (53.1%)	
squamous-cell carcinoma	26 (36.1%)	20 (40.8%)	
Baseline multiple-metastases sites, n (%)			0.713
No	32 (44.4%)	20 (40.8%)	
Yes	40 (55.6%)	29 (59.2%)	
Baseline lymph node stage, n (%)			0.572
N1/2	43 (59.7%)	32 (65.3%)	
N3	29 (40.3%)	17 (34.7%)	
ICI therapy regimen, n (%)			0.409
ICI + chemotherapy	55 (76.4%)	34 (69.4%)	
ICI	17 (23.6%)	15 (30.6%)	
Clinical outcome, n (%)			0.026
DCB	46 (63.9%)	21 (42.9%)	
NDB	26 (36.1%)	28 (57.1%)	
TMB level, n (%)			0.013
<10 mut/mb	47 (65.3%)	42 (85.7%)	
≥10 mut/mb	25 (34.7%)	7 (14.3%)	

N: lymph node; ICI: immune checkpoint inhibitors; DCB: durable clinical benefit; NDB: no durable benefit; TMB: tumor mutation burden; mut/mb: mutations per megabase; DNA damage response: DDR.

**Table 2:** Frequency of DDR gene alterations in NSCLC patients with PD-L1 high expression.

Gene Class	Gene	DCB	NDB
Base excision repair	*MUTYH*	2 (2.7%)	0 (0%)
	*PARP1*	3 (4.2%)	0 (0%)
Damage sensor	*ATM*	4 (5.6%)	3 (6.1%)
	*ATR*	8 (11.1%)	11 (22.4%)
	*CHEK1*	0 (0%)	1 (2.0%)
	*CHEK2*	1 (1.4%)	3 (6.1%)
Fanconi anaemia	*FANCA*	8 (11.1%)	1 (2.0%)
	*FANCC*	2 (2.7%)	0 (0%)
	*FANCG*	0 (0%)	1 (2.0%)
	*FANCL*	1 (1.4%)	0 (0%)
	*XRCC2*	3 (4.2%)	0 (0%)
Homologous recombination	*BARD1*	0 (0%)	1 (2.0%)
	*BRCA1*	5 (6.9%)	3 (6.1%)
	*BRCA2*	4 (5.6%)	4 (8.2%)
	*BRIP1*	1 (1.4%)	0 (0%)
	*NBN*	1 (1.4%)	1 (2.0%)
	*PALB2*	6 (8.3%)	1 (2.0%)
	*RAD51*	1 (1.4%)	1 (2.0%)
	*RAD51B*	2 (2.7%)	1 (2.0%)
	*RAD51C*	0 (0%)	0 (0%)
	*RAD51D*	0 (0%)	0 (0%)
	*RAD52*	0 (0%)	1 (2.0%)
	*RAD54L*	0 (0%)	2 (4.1%)
Mismatch repair	*MLH1*	1 (1.4%)	1 (2.0%)
	*MSH2*	3 (4.2%)	1 (2.0%)
	*MSH3*	5 (6.9%)	0 (0%)
	*MSH6*	2 (2.7%)	1 (2.0%)
	*PMS2*	4 (5.6%)	1 (2.0%)
Nucleotide excision repair	*ERCC4*	5 (6.9%)	1 (2.0%)
	*POLE*	2 (2.7%)	2 (4.1%)
SWI/SNF chromatin remodelling	*ARID1A*	13 (18.1%)	2 (4.1%)
	*SMARCA4*	7 (9.7%)	4 (8.2%)
TP53 pathway	*MDM2*	1 (1.4%)	0 (0%)
	*MDM4*	1 (1.4%)	0 (0%)
	*TP53*	46 (63.9%)	36 (73.5%)

SWI/SNF, SWItch/Sucrose Non-Fermentable chromatin remodelling complex; *MUTYH*: mutY DNA glycosylase; *PARP1*: poly(ADP-ribose) polymerase 1; *ATM*: ataxia telangiectasia mutated; *ATR*: ataxia telangiectasia and Rad3 related; *CHEK1*: checkpoint kinase 1; *CHEK2*: checkpoint kinase 2; *FANCA*: Fanconi anemia complementation group A protein; *FANCC*: Fanconi anemia complementation group C protein; *FANCG*: Fanconi anemia complementation group G protein; *FANCL*: Fanconi anemia complementation group L protein; *XRCC2*: X-ray repair cross-complementing protein 2; *BARD1*: BRCA1 associated RING domain 1; *BRCA1*: BRCA1 DNA repair associated; *BRCA2*: BRCA2 DNA repair associated; *BRIP1*: BRCA1 interacting helicase 1; *NBN*: nibrin; *PALB2*: partner and localizer of BRCA2; *RAD51*: RAD51 recombinase; *RAD51B*: RAD51 paralog B; *RAD51C*: RAD51 paralog C; *RAD51D*: RAD51 paralog D; *RAD52*: RAD52 homolog DNA repair protein; *RAD54L*: RAD54 like helicase; *MLH1*: mutL homolog 1; *MSH2*: mutS homolog 2; *MSH3*: mutS homolog 3; *MSH6*: mutS homolog 6; *PMS2*: postmeiotic segregation increased 2; *ERCC4*: excision repair cross-complementation group 4; *POLE*: DNA polymerase epsilon catalytic subunit A; *ARID1A*: AT-rich interaction domain 1A; *SMARCA4*: SWI/SNF related matrix associated actin dependent regulator of chromatin subfamily A member 4; *MDM2*: mouse double minute 2 homolog; *MDM4*: mouse double minute 4 homolog; *TP53*: tumor protein p53; DCB: durable clinical benefit; NDB: no durable benefit.

## Data Availability

The datasets generated and/or analyzed during the current study are not publicly available due to ethical and privacy restrictions regarding patient-level clinical and genomic data. De-identified data may be available from the corresponding author upon reasonable request and with approval from the institutional ethics committee.

## Supplementary Materials

The following supporting information can be downloaded at https://www.mdpi.com/article/10.3390/curroncol33090508/s1, Figure S1: Multivariable Cox regression analysis of PFS. Forest plot showing HRs and 95% CIs for factors associated with PFS. Multivariable Cox regression analysis was adjusted for DDR status, TMB, treatment regimen, age, ECOG performance status, smoking history, and PD-L1 expression level. DDR-positive status remained independently associated with prolonged PFS (HR = 0.54, 95% CI: 0.31–0.94, *p* = 0.020), whereas TMB was not significantly associated with PFS. HRs were estimated using the Cox proportional hazards model, with progression or death as the event.
