# Supplementary material for: DNA Damage Response Alterations Stratify Response to ICI-Based Therapy in Advanced NSCLC with High PD-L1 Expression
Source: Curr Oncol. 2026 Aug 26;33(9):508. doi: 10.3390/curroncol33090508 (PMC13605398; doi:10.3390/curroncol33090508)
Supplement: Supplementary file 1 [file curroncol-33-00508-s001.zip › curroncol-4415075-supplementary.pdf]

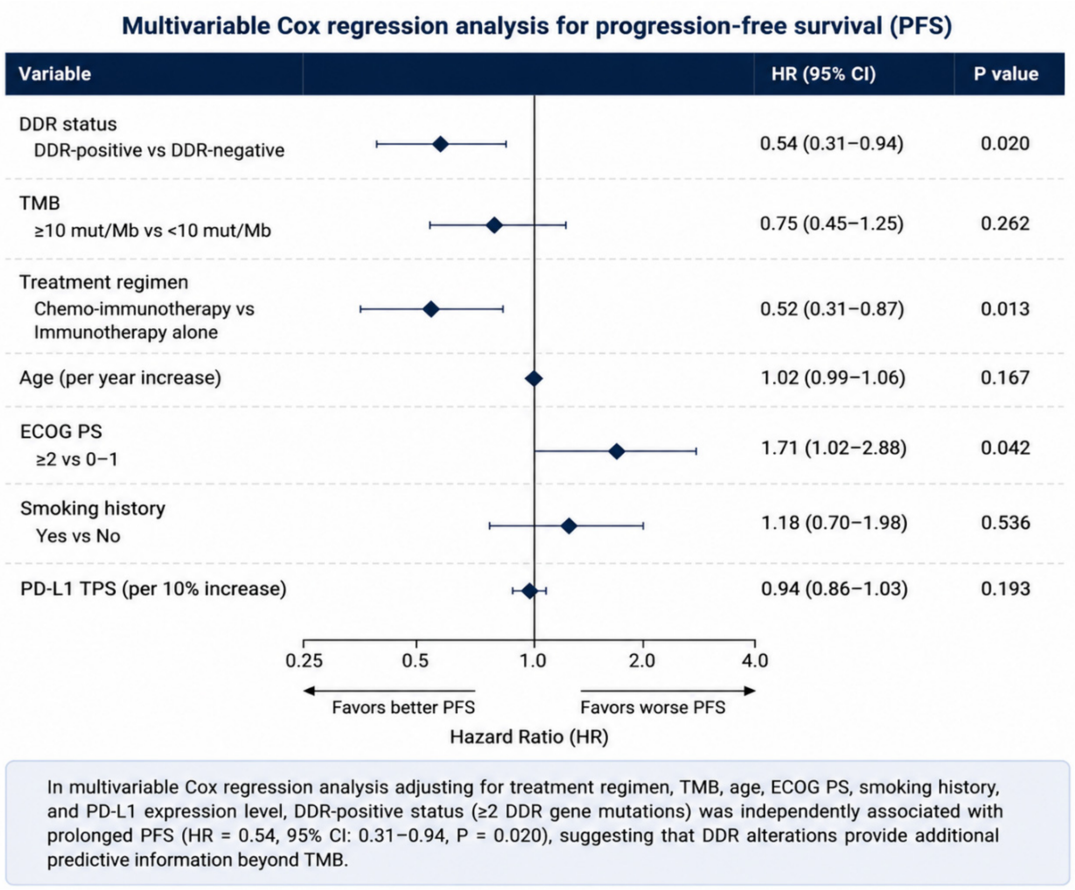

Figure S1. Multivariable Cox regression analysis of PFS. Forest plot showing HRs and 95% CIs for factors associated with PFS. Multivariable Cox regression analysis was adjusted for DDR status, TMB, treatment regimen, age, ECOG performance status, smoking history, and PD-L1 expression level. DDR-positive status remained independently associated with prolonged PFS (HR=0.54, 95% CI: 0.31-0.94, *p*=0.020), whereas TMB was not significantly associated with PFS. HRs were estimated using the Cox proportional hazards model, with progression or death as the event.
